# Integrating the glioblastoma microenvironment into engineered experimental models

**DOI:** 10.4155/fsoa-2016-0094

**Published:** 2017-03-24

**Authors:** Weikun Xiao, Alireza Sohrabi, Stephanie K Seidlits

**Affiliations:** 1 Department of Bioengineering, University of California, Los Angeles, CA 90095-1600, USA

**Keywords:** biomaterials, extracellular matrix, glioblastoma, microenvironment

## Abstract

Glioblastoma (GBM) is the most lethal cancer originating in the brain. Its high mortality rate has been attributed to therapeutic resistance and rapid, diffuse invasion – both of which are strongly influenced by the unique microenvironment. Thus, there is a need to develop new models that mimic individual microenvironmental features and are able to provide clinically relevant data. Current understanding of the effects of the microenvironment on GBM progression, established experimental models of GBM and recent developments using bioengineered microenvironments as *ex vivo* experimental platforms that mimic the biochemical and physical properties of GBM tumors are discussed.

## Abstract (lay abstract)

Glioblastoma (GBM), the most common form of brain cancer, is extremely lethal due to its rapid progression, resistance to treatment and high rates of recurrence. As reviewed here, the microenvironment that surrounds GBM tumors drives their progression. Development of effective treatments for GBM will require better experimental models that account for the tumor microenvironment and thus, can provide clinically translatable results. This review focuses on using biomaterials to create tumor-mimetic microenvironments as experimental platforms for cell culture.

Glioblastoma (GBM), or grade IV glioma, is an extremely lethal cancer originating in the brain with a median survival time of only 12–15 months [1]. GBM tumors aggressively infiltrate the brain, preventing complete surgical resection and overwhelmingly acquire resistance to chemotherapy and radiation, leading to inevitable recurrence. GBM cells dynamically respond to their local tissue microenvironment, which, in turn, plays a critical role in tumor invasion and treatment resistance [2–4]. Although various microenvironmental features strongly influence GBM physiology, current models fail to account for the complex microenvironment surrounding GBM tumors and do not adequately reflect clinical outcomes. Development of effective treatments will require advanced experimental tools that more accurately model clinical physiology. Here, we review commonly used experimental models of GBM, recent improvements to these models and strategies for developing advanced models. Particular attention is given to bioengineered models that use biomaterials to mimic the chemical and physical properties of the GBM microenvironment.

Experimentally it has been challenging to isolate the influence of any individual feature of the complex GBM microenvironment on tumor physiology. Bioengineered platforms enabling modular control over these independent variables can potentially isolate these effects and identify new therapeutic targets. In the first part of this review, we discuss individual features of the microenvironment separately as independent variables affecting GBM physiology. In the second part, we discuss how biomaterials might be engineered to create complex models of this microenvironment in which both the integrated and decoupled effects of each feature can be robustly characterized. While researchers have made some progress controlling multiple aspects of the tumor microenvironment – for example, extracellular matrix (ECM), soluble biomolecule signals, physical properties and cell–cell interactions – within bioengineered microenvironments, major advancements to date have centered around methods to orthogonally control individual biochemical and biophysical features of the ECM. Thus, while other aspects of the GBM microenvironment are included, the second part of this review focuses on the use of 3D biomaterials to model ECM-related features.

## The unique brain microenvironment drives GBM progression

**Figure 1.**
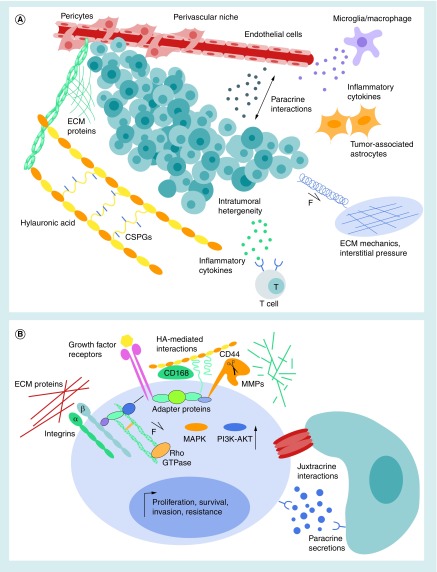
**Complex microenvironment surrounding glioblastoma tumors.** **(A)** GBM microenvironment at the tissue scale. HA, glycosaminoglycans, proteoglycans and proteins in the ECM relay mechanical and biochemical cues to tumor cells. An increase in interstitial pressure in the tumors also contributes to the mechanical microenvironment. GBM tumors are made up of a heterogeneous mixture of cells with different phenotypes, including stem-like cells. Other tumor-supportive cells in the microenvironment include those in the perivascular niche (endothelial cells and pericytes), astrocytes and immune cells (microglia/macrophages and T cells). **(B)** Microenvironmental features at the level of single GBM cells. Adhesion to ECM proteins through integrins relay biochemical and mechanical cues through the actin cytoskeleton and intracellular signaling pathways, including Rho-GTPase. CD44 and CD168, mediate interactions with HA in the surrounding ECM. Growth factor binding activates receptors, including tyrosine kinases that upregulate oncogenic MAPK and PI3K/AKT pathways. Growth factor receptors, HA receptors and integrins interact through membrane-associated adapter proteins to amplify oncogenic pathways through feedback loops. Membrane-bound MMPs anchor to CD44 to facilitate ECM degradation and cell invasion. Cell–cell interactions occur directly through gap or cadherin-mediated adherens junctions (juxtracrine interactions) and indirectly through secreted soluble factors (paracrine interactions). Together, GBM cells integrate these microenvironmental cues, resulting in upregulation of genes promoting survival, proliferation and treatment resistance. ECM: Extracellular matrix; GBM: Glioblastoma; HA: Hyaluronic acid; MMP: Matrix metalloprotease.

Isolated behind the blood–brain barrier (BBB), the brain microenvironment is distinct from that in peripheral tissues. Originating in the brain, GBM tumors closely interact with this unique microenvironment [4]. Even highly aggressive tumors rarely metastasize outside of the brain [5], indicating a preference for the brain microenvironment. In contrast, tumors originating outside of the CNS that metastasizes to the brain are typically less integrated with the local ECM and only invade short distances [3]. Comprising around 20% of the tissue volume, the brain ECM contains few fibrous proteins and high amounts of specialized proteoglycans (PGs), glycosaminoglycans (GAGs) and glycoproteins [3–4,6]. Cell–cell interactions, tissue mechanics and the presence of soluble cytokines, growth factors and gases (e.g., oxygen and nitric oxide) also comprise the microenvironment – presenting a complex landscape which is altered in the presence of GBM tumors to support cancer invasion and treatment resistance. Figure 1 demonstrates how many microenvironmental cues act in tandem to shape the pathological phenotype of GBM at the levels of the entire tumor tissue (Figure 1A) and individual cancer cells (Figure 1B). Moreover, the relationship between GBM tumors and their microenvironment is highly dynamic and reciprocal. For example, GBM cells excessively secrete ECM, triggering a positive feedback through both mechanically and chemically induced cell receptors, which further upregulates the expression of ECM components, receptors and ECM-degrading enzymes. For detailed reviews on the GBM microenvironment, please refer to [3,4,7],^4^.

### ECM composition

Hyaluronic acid (HA) – a negatively charged, unbranched GAG – is highly abundant in the brain ECM [3,4]. In healthy brain, high-molecular-weight (>10^6^ Da) HA chains act as the organizational center of the ECM, interacting with proteins and PGs through small linker proteins, known as HABPs, to create a hydrogel-like mesh [8]. HA is upregulated in GBM tumors where it contributes to many phenotypic changes associated with cancer progression including initial tumor development, cancer cell proliferation, invasion, resistance to therapeutic agents and post-treatment recurrence [3–4,6,9–12]. In addition to HA, HA synthases, hyaluronidases, HA receptors and some HABPs are overexpressed [13–15]. Concurrent overexpression of these factors likely contributes to the hyperaggression and treatment resistance in GBM.

HA interactions with the CD44 and CD168 (aka., the RHAMM) receptors further promote growth, invasion and treatment resistance in GBM and many other cancers [6,11,14,16]. HA–CD44 binding upregulates PI3K–AKT and MAPK–ERK1/2 signaling pathways, resulting in increased apoptotic resistance and migratory capacity [6,17–18]. Although the complex mechanisms driving HA-mediated drug resistance are not fully understood, disruption of HA–CD44 interactions decreases invasive potential and increases susceptibility to drug-induced apoptosis [6,10,12,16]. Moreover, HA-bound CD44 interacts with several other membrane-associated proteins, including tyrosine kinases, matrix metalloproteases (MMPs), integrins and drug efflux transporters, reinforcing activities that drive cancer progression [11,17–20].

Overexpression of the product of the *MDR1* gene – P-gp, a cell membrane transporter involved in drug efflux – in GBM and many other cancers is associated with increased resistance to chemotherapy and radiation [6,11]. CD44 and P-gp both anchor to the cytoskeleton and closely associate with lipid microdomains in the cell membrane where their interactions increase and stabilize *MDR1* expression [11,19]. HA–CD44 and HA–CD168 interactions enhance cell motility [4,10,20]. Aggressive invasion is common to all GBM tumors, regardless of molecular subtype [21]. HA-facilitated migration may partially explain why GBM invasion is concentration near HA-rich vasculature, white matter tracts and the rostral migratory stream in the brain [21–27]; please refer to [12] for a thorough review HA in GBM and [21] for an extensive review on GBM invasion.

Cell attachment to ECM proteins is typically mediated by membrane-spanning integrin receptors. HA-bound CD44 receptors act synergistically with engaged integrins to promote cell migration [18,20,28]. Several integrins (e.g., β_1_, β_3_, β_5_ and α_v_) are overexpressed by GBM cells [27,29–30]. As HA alone does not typically support cell adhesion and migration, additional integrin-binding proteins are required [20,28]. Increased deposition of several ECM proteins during GBM progression, including vitronectin, tenascin-C, osteopontin and osteonectin, directly correlates with poor prognosis and invasion [3–4,7,12,14]. The majority of these upregulated ECM proteins contain the universal integrin-binding sequence, RGD. ECM binding to GBM cell integrins generally leads to increased apoptotic resistance, proliferation and migration [27,30]. For example, GBM cell invasion along microvasculature is likely facilitated through integrin – likely α_3_β_1_ – interactions with collagen IV and laminin [24,31]. For a detailed review of integrins as targets for GBM therapies, please refer to [30].

Glycoproteins (e.g., tenascin-C), and chondroitin sulfate and heparan sulfate PGs (e.g., versican) are also upregulated around GBM tumors [3,32]. Glycosylated proteins are involved in a wide range of functions, ranging from cell migration to growth factors [32]. In particular, heparan sulfate facilitates the activation of oncogenic tyrosine kinase receptors via sequestration of growth factors, including EGF, PDGF-A and TGF-β [32–34]. Versican interactions with TGF-β promote tumor cell migration [32]. Effects of PGs on GBM cells often depend on the presence of other ECM components. For example, one study reported that the chondroitin sulfate PG brevican is cleaved by migrating GBM cells (including several transformed and patient-derived GBM lines) and that this cleavage product associates with fibronectin to further promote invasion [35]. Despite a few isolated studies, the function of PGs in GBM progression remains largely unknown [32]. In reality, it is likely that complex interactions between PGs, GAGs and other ECM proteins ultimately dictate GBM physiology in a way that is unique from the effects of any individual ECM component.

### Soluble factors in the extracellular space

Several bioactive, cell-produced soluble factors are also abundant in GBM microenvironment. Tumor-associated overexpressions of TGF-β, TGF-α, EGF, VEGF and TNF-α promote GBM cell survival and proliferation [36,37]. Thus, therapies targeting TGF-β, EGF and VEGF have all been investigated in clinical trials [36]. More than 50% of GBM tumors bear amplification and/or mutation of the EGFR, while around 11% overexpress receptors for PDGF (PDGFR) [38]. GBM cell overexpression of PDGF-A triggers an autocrine loop that promotes GBM proliferation and survival [39]. EGFR-dependent tumors typically acquire resistance to pharmaceutical inhibition, often by switching growth dependence to PDGFR pathways [38]. Together, heparin-bound EGF and TGF-α participate in an autocrine loop to further amplify oncogenic EGFR signaling and promote GBM invasion [36,40–41]. TGF-α may also play an important role in GBM initiation, as it promotes conversion of mature astrocytes to neural progenitor-like phenotypes [42]. Tyrosine kinase receptors, including those for EGF, TGF-α and PDGF-A, also interact with ECM receptors to increase tumor progression [18,20,33,43–45]. For example, CD44 localizes near EGFR to augment activation of ERK1/2-MAPK and PI3K–AKT pathways, increasing GBM cell migration and apoptotic resistance [18,40]. The chemoattractant CXCL12 (aka., SDF-1), produced by GBM-tumor-associated microglia/macrophages and endothelial cells, also promotes GBM invasion through interactions with CXCR4 [46–48].

Abnormal profiles of inflammatory cytokines in the GBM microenvironment contribute to increased invasion, angiogenesis and other pathological characteristics [37,49]. Widely studied are the effects of TGF-β, which promotes GBM proliferation (by increasing PDGF-B production [50]), angiogenesis (by upregulating VEGF [51] and tumor invasion (by enhancing MMP expression) [52]. TGF-β also inhibits tumor clearance by cytotoxic T cells [53] and induces infiltrating macrophages and microglia to adopt a proinflammatory phenotype, known as M1 [52,54]. While proinflammatory, M1-type macrophages support GBM growth, conversion to proresolving, M2-type macrophages appear to delay growth [55]. Similar to TGF-β, the expression of proinflammatory TNF-α induces macrophages to exhibit M1-type characteristics [54]. TNF-α activates a feed-forward loop – inducing a TLR4-dependent upregulation of AKT and HIF-1α that sustains the inflammatory response in GBM [56]. TNF-α also enhances tumor angiogenesis through increasing production of VEGF and basic FGF-2 [57].

Low-molecular-weight HA chains also act as potent soluble factors in the GBM microenvironment [11]. Unlike the high-molecular-weight form, low-molecular-weight HA can activate TLRs on immune cells to act as potent proinflammatory factors [58]. For example, low-molecular-weight HA activates TLR4 and induces TNF-α expression in macrophages [11,59]. Given these observations, it is like that low-molecular-weight HA is another participant in the proinflammatory TNF-α autocrine loop described above. In cases of chronic inflammation, including GBM, high-molecular-weight HA in the ECM is degraded to smaller fragments by overexpressed hyaluronidases, which can drive cancer progression, promoting angiogenesis and invasion [26,60]. Wu *et al*. recently provided clinical evidence relating HA degradation and cancer progression by demonstrating that serum levels of low-molecular-weight HA (<50 kDa) in breast cancer patients, but not levels of total HA, correlate positively with occurrence of lymph node metastasis [61]. Despite these findings, the molecular-weight-dependent effects of HA in GBM remain unclear. Thus, future models of the GBM microenvironment would benefit from accounting for these effects. For example, cell-mediated degradation of biomaterials fabricated from high-molecular-weight HA into fragments, the diffusion of these fragments, and their effects on cultured GBM cells, could all be monitored. For more detailed reviews of cytokines and other soluble factors in the GBM microenvironment, please refer to [11,36–37].

### ECM degradation

In addition to forming adhesive interactions with the ECM, GBM and other tumor-associated cells produce several ECM-degrading enzymes that facilitate invasion throughout the brain [62–64]. Matrix remodeling is also necessary for angiogenesis, which acts to ‘feed’ tumors [62,65]. In GBMs, overexpression of MMP-2 and MMP-9 correlates with poor survival [66,67]. MMP-2 targets several ECM proteins found in the brain, including various types of collagen, fibronectin, laminin, MBP, osteonectin (also known as SPARC), tenascins and vitronectin [62]. MMP-9 degrades laminin, osteonectin and vitronectin; however, it has a high affinity for collagen IV – a basement membrane component concentrated near blood vessels [62]. Inhibition of MMP-2 or MMP-9 activity reduces glioma invasion and growth in experimental models [65,68].

Effects of MMPs on GBM progression are amplified through interactions with several other features in the GBM microenvironment, including cell receptors, ECM components and soluble factors. For example, MMP-2 physically localizes with integrin α_v_β_3_ – which mediates adhesion to vitronectin to promote cell migration near blood vessels [69] – on the cell surface to further enhance migration [65]. Similarly, CD44 acts as an anchor for MMP-9, facilitating degradation of the HA-rich matrix [70]. HA also induces MMP-9 overexpression in GBM cells with loss of phosphate and tensin homolog function – a common clinical mutation in GBM [43]. Finally, MMP degradation releases matrix-bound soluble factors promoting invasion, including TGF-β [70,71].

In addition to MMPs, hyaluronidases, plasminogen activators and ADAMs facilitate GBM invasion through matrix degradation. In several cancers, hyaluronidase overexpression is associated with malignancy and increased aggression [72]. Hyaluronidase degradation of high-molecular-weight HA creates low-molecular-weight HA fragments, which can induce angiogenesis and inflammation near the tumor, as described in the previous section [11,73]. Plasminogen activators, also overexpressed in GBM, have likewise been implicated in tumor invasion [63] and angiogenesis [74]. Finally, ADAM proteases, and, in particular, ADAM-10 overexpression, has been correlated to upregulation of MMP-2 and MMP-9 in GBM tumors [75].

### Mechanical properties

Cells sense and respond to micron-scale gradients of mechanical rigidity throughout the brain [76]. Mechanical signals are transduced by multiple receptors, including integrins, G-protein-coupled receptors, stretch-activated ion channels and CD44 [20,28,77–78]. Several studies have confirmed integrin-mediated activation of the Rho-family GTPases, FAK/PI3K/AKT and ERK/MAPK pathways – each of which is upregulated in migrating cells – in response to mechanical cues [20–21,28,77]. In experimental GBM models, an integrin-mediated positive feedback loop has been identified where migrating cells stiffen their surrounding matrix, which, in turn, increases motility [20,28]. Receptors anchored to the actin cytoskeleton, including integrins and CD44, can rapidly relay mechanical stimuli through release of actin-bound transcription factors and direct coupling to the nuclear membrane [79]. Tyrosine kinase receptors also respond to mechanical cues in the tumor microenvironment [77,80]. For example, in airway epithelial cells, application of compressive stress triggers increased EGFR phosphorylation followed by downstream amplification of ERK activity [81].

Mechanical cues and tumor stiffening have marked influences on drug response and invasion of several types of cancer [77,82]. Several studies have demonstrated that GBM cells respond to mechanical signals [80,83–86]. Although many researchers have posited that GBM tumor tissues are stiffer than healthy brain, conflicting data are reported depending on the tumor source and measurement method. For example, while one study found linear compressive moduli of xenografted tumors *ex vivo* to be at least 20-times stiffer than normal mouse brain [87], a later study found no differences in shear moduli [88]. In clinical patients, ultrasound-based shear wave elastography measurements found GBM tumors to have approximately twice the Young's moduli of surrounding normal brain tissue [89].

Although studies of GBM tissue agree that stiffness increases, it is unclear whether this increase can be attributed to changes in compliance with the ECM, the cells themselves, increased interstitial pressure or a combination of these variables. While no reports of GBM cell stiffness were found at the time of this review, in many cancers of peripheral origin, cells are softer than their healthy counterparts [90]. Thus, it is likely that GBM cells also become softer than normal brain cells. This mechanical shift is thought to facilitate cell invasion [91] and enable oncogenic changes in gene expression [92].

As the GBM tumor grows, the local interstitial pressure rises [93,94]. While cerebral spinal fluid normally drains through the perivascular lymphatic system and tissue stroma [95], fluid accumulates in GBM tumors, resulting in a sharp gradient of increased interstitial pressure between the tumor and healthy tissues [93]. Increases in fluidic pressure activate similar mechanotransduction pathways as ECM stiffening [88]. In GBM, fluid accumulation results in the enlargement of the extracellular space and slower diffusion through the tissue, despite the increased cell density and ECM deposition [4,87]. This seemingly paradoxical observation may be explained by GAG deposition – charged GAGs are known to not only take up water but also to bind and sequester diffusing ions and even growth factors [4,32,70]. Recent studies have demonstrated that feedback from this buildup of interstitial pressure drives GBM tumor growth and invasion [96,97]. Specifically, this pressure-driven invasion appears to be mediated by the CXCL12-driven chemotaxis (through CXCR4) and HA–CD44 interactions [97].

### Homocellular & heterocellular interactions

Direct homocellular interactions between GBM cells through gap junctions convey protection from drug-induced apoptosis [98]. Specifically, knockdown of the gap junction protein connexin-43 sensitizes cells to temozolomide chemotherapy, a common clinical treatment for GBM. Homotypic interactions between GBM cells are also mediated by cadherins, but their exact role in GBM remains unclear, largely due to conflicted reports. Several reports have demonstrated that GBM cells lacking N-cadherin-based adherens junctions are more invasive [99,100]. However, others have reported that N-cadherins are upregulated in GBM and do not hinder GBM invasion [101]. In a recent study, researchers found that cell–cell interactions through N-cadherins activate β-catenin, and subsequently the canonical Wnt pathway promotes GBM invasion and drug resistance [102].

Over 10 years ago, researchers identified and isolated a subpopulation of GBM cells commonly known as glioblastoma stem cells (GSCs) [103]. Although relatively few in number, GSCs are thought to strongly contribute to tumor initiation, invasion, therapeutic resistance and recurrence [103,104]. Collectively, these observations have generated the theory that treatment-resistant GSCs become the dominant population in recurrent tumors [105]. For detailed reviews of GSCs and their microenvironmental niche, please refer to [106,107]. GSC function depends on their microenvironment, including direct interactions with non-GSC tumor cells and noncancerous cells [104]. GSCs also interact with adjacent cells through gap and adherens junctions. While non-GSC GBM cells express higher levels of connexin-43, GSCs upregulate another gap junction protein, connexin-46, which is essential for their self-renewal and maintenance [106,108]. N-cadherin may also facilitate GSC invasion via integrin α_6_ [109].

To satisfy their high nutritional and oxygen demands, GBM tumors utilize several mechanisms of neovascularization, including vascular co-option (tumor hijacking of normal vessels), angiogenesis (sprouting of new vessels) and vascular mimicry (formation of vessel-like structures by tumor cells) [110]. GBM tumors secrete angiogenic growth factors, such as VEGF, to recruit pericytes and endothelial cells [36,52]. GBM cells can also directly interact with pericytes through Cdc42- and actin-based extensions to modify contractile activity of pericytes [111]. Notably, GBM cells can transdifferentiate into endothelial cells [112] or pericytes [113] to effectively create new vessels.

In addition to direct provision of nutrients, a large body of evidence suggests that the perivascular niche acts to maintain the ability of GSCs to induce tumor initiation and therapeutic resistance [2,114–115]. Migrating GSCs interact with the surface of existing blood vessels to facilitate their invasion throughout the brain parenchyma [116]. Direct interactions between integrin α_5_ on GBM cells and laminin-α2 – enriched near brain tumor vessels – critically regulate GSC maintenance and growth [117,118]. In addition to the perivascular niche, a hypoxic niche is important for GSC maintenance [119]. GBM tumors grow rapidly and vascularization often lags behind [104], creating a hypoxic gradient that induces cells farthest from blood vessels to upregulate HIF transcription factors [58,119]. The downstream targets of HIFs, including *Oct4*, *Sox2*, *LOX* and *CXCR4*, then maintain the GSC function. HIFs also induce secretion of GBM-supportive growth factors, including PDGF-B and VEGF [120].

GBM cells compromise normal interactions between nontumor cells. In particular, they disrupt the BBB through both secretion of soluble factors [116] and direct physical displacement of astrocytic endfeet from the vascular surface [31]. Accumulation of such ‘leaky’ vessels around the GBM tumor subsequently contributes to increased interstitial pressure [94]. However, a leaky BBB may be an advantage for treatment, where small molecule drugs that would normally be rejected by an intact BBB could more efficiently cross and reach tumors. GBM cell–astrocyte interactions mutually support survival of both cell types [121,122]. In cultures, GBM-associated astrocytes adopt an inflammatory, reactive phenotype exemplified by elevated expression of GFAP [99]. Furthermore, in cell culture, chemokines secreted by astrocytes have been reported to induce GSCs to adopt a more invasive phenotype [121].

In healthy CNS, microglia act as the primary macrophage-like immune cells. However, in the GBM microenvironment, blood monocyte-derived macrophages, which have crossed the BBB through leaky vessels, are also present [123]. Together, microglia and macrophages comprise a significant portion of GBM tumor-associated cells (possibly up to 50%) and provide an immunosuppressive environment that facilitates cancer progression [55,64,123]. Microglia/macrophages are recruited tumors by cytokines in the GBM microenvironment; for example, by the potent immune cell chemoattractant CXCL12 [46,123]. M1-type microglia/macrophages also produce TGF-β and MMPs, promoting GBM cell invasion and tumor growth [71,124]. Interestingly, migrating microglia secrete MMP-2 to degrade pathways through the brain parenchyma, which are subsequently hijacked by invading GBM cells [125].

## Currently used experimental models of GBM

**Figure 2.**
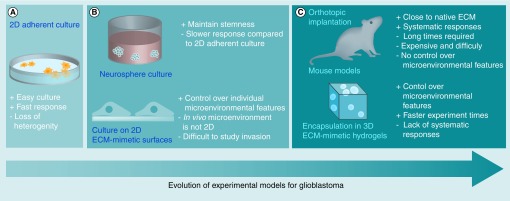
**Advancements in experimental models of glioblastoma tumors.** **(A)** 2D monolayer cultures on protein-coated plastic or glass. **(B)** Suspension culture of patient-derived neurospheres (top) and 2D culture on biomimetic materials (bottom). **(C)** Orthotopic transplantation of patient-derived cells into mice (top) and 3D culture of glioblastoma cells in biomaterial microenvironments (bottom). ECM: Extracellular matrix.

Multiple *in vitro* and *in vivo* models have been widely used to study GBM physiology and evaluate the therapeutic efficacy of potential clinical treatments. This section discusses advantages and disadvantages of common experimental models with particular emphasis on their ability to provide clinically translatable results (Figure 2). Moreover, we discuss the capacity of each model to account for and identify individual aspects of the GBM microenvironment, which dictate tumor progression and treatment resistance.

### 2D culture models

Since the 1960s and 1970s, researchers have used 2D monolayer cultures of cells, derived clonally from patient tumors, to study GBM physiology (Figure 2A) [126]. Of these lines, U87MG has been widely used to collect data for scientific publications [127]. Despite extensive data collected using these lines, it is difficult to interpret their clinical relevance given occurrence of mutations and ‘phenotypic drift’ since isolation of the original cells, and inadequate representation of heterogeneity seen in clinical GBM.

Serum-based, monolayer cultures have been widely used to evaluate effects of various agents on GBM cells. Serum contains various ECM proteins and soluble factors that can aid in cell adhesion to culture substrates and promote proliferation of cultured cells. However, GBM cell cultures reliant on serum-containing medium have many disadvantages. First, serum induces selection of cells with specific characteristics, reducing culture heterogeneity with subsequent passages [128]. Second, serum can cause significant phenotypic and genotypic changes in cultured GBM cells [128,129]. Finally, serum is derived from animal sources (often bovine) suffers from poor batch-to-batch reproducibility [130]. Taken together, these effects may explain why many treatments that are successful *in vitro* fail in clinical trials.

Substrates for monolayer cultures are often coated with ECM proteins to facilitate cell adhesion and/or study the GBM cell–ECM interactions. However, adsorption to glass or plastic substrates effectively denatures the protein, making the *in vivo* relevance of experimental results difficult to discern. In addition, it is difficult to recapitulate the complex mixture of proteins in the native GBM microenvironment by coating a 2D culture substrate. To better mimic this mixture of ECM components, Matrigel^®^ has been widely used. Matrigel is derived from the basement membrane of Engelbreth–Holm–Swarm mouse sarcomas and, according to the manufacturer Corning^®^ Life Sciences (Corning, NY, USA), contains approximately 60% laminin, 30% collagen IV and 8% entactin as well as several bound growth factors, including EGF, TGF-β and PDGF. Matrigel has the advantage of compatibility with 2D or 3D cell culture. However, although this protein mixture may be relevant to some peripheral cancers, it does not accurately reflect the GBM microenvironment. Finally, it is difficult to study the effects of individual ECM components and soluble factors using Matrigel, as their composition cannot be experimentally controlled. Similar to serum reagents, lot-to-lot variability and derivation from a nonhuman source are also major concerns.

### Suspension-based culture models

More recent efforts have developed methods for GBM cell isolation and culture that can generate data with better relevance to clinical outcomes [128,131]. To maintain GSC-like and patient-specific behaviors, tumor cells are dissociated from freshly isolated patient biopsies and cultured in suspension as clonally dividing neurospheres (also known as gliomaspheres) in serum-free, xeno-free medium supplemented with EGF and basic FGF-2 (Figure 2B) [127,130]. Unlike serum-cultured glioma lines, neurosphere cultures derived from human GBM tumors better preserve the genotypic, phenotypic and *in vivo* characteristics of the original clinical patient [128,131–132]. In addition to these advantages, neurosphere cultures provide a semi-3D environment in which cells deposit ECM to create their own unique microenvironment [132]. Neurosphere cultures have enabled many important findings, including characterization of GSC microenvironmental niches [2] and clinically relevant mechanisms of treatment resistance [131].

Using data from The Cancer Genome Atlas, GBM tumors have been classified by gene expression into four clinically relevant subtypes: classical, proneural, neural and mesenchymal [133]. Neurosphere lines isolated from primary patients’ samples representing different GBM tumor subtypes have facilitated the understanding of the differences between the subtypes and, in particular, differences in disease progression and treatment efficacy. Studies comparing mesenchymal GBM-derived neurospheres with those isolated from tumors of other subtypes may provide insights into the mechanisms of an epithelial-to-mesenchymal like process in GBM [134]. In many cancers, upregulation of mesenchymal genes, as embodied by the mesenchymal GBM subtype, represents a transition to a more aggressive stage of the disease with increased tumor invasion and treatment resistance [135]. Given that different GBM subtypes express distinct receptors for microenvironmental features, such as EGFR, PDGFR and CD44, it is not unlikely that these tumors also contain microenvironments unique to each subtype. Finally, while these four subtype classifications appear to fully capture primary GBM tumors [133], these characteristics likely exist along a continuum in patients and change in recurrent, secondary tumors [135].

Despite improvements over monolayer, GBM cell line cultures, neurosphere cultures do not adequately capture all aspects of GBM tumors. First, neurospheres are highly enriched in GSC-like cells, which reside in relatively low abundance in native tumors [106], while cells of various subtypes within the original tumor population with low self-renewal capabilities are lost [128,136]. Furthermore, neurosphere formation is a strictly *in vitro* phenomenon. In contrast, GSCs *in vivo* typically reside in a perivascular niche where they experience a different microenvironment [2].

Recently, Hubert *et al*. [137] generated 3D GBM organoids that reached several millimeters in diameter – much larger than typical neurospheres which are on the order of 100–200 μm. Hypoxic gradients were present throughout the organoid, inducing phenotypic differences in cells residing in the periphery or at the core reminiscent of those seen in human tumors. Organoids contained radiation-resistant GSCs in the core, indicating that this model better captures how tumor heterogeneity contributes to treatment resistance and recurrence. Finally, when orthotopically transplanted in mice, organoids formed tumors with histological features better resembling clinical GBM tumors than did patient-matched neurospheres. While organoid cultures provide an *ex vivo* model of GBM that better mimics heterogeneity, treatment resistance and invasion of clinical tumors than other culture systems, organoid generation requires months of culture while neurosphere cultures take only weeks.

### *In vivo* rodent models

Although *in vitro* models present the opportunity to perform more experiments within a shorter period of time, they do not account for many of the microenvironmental influences – for example, GBM–stromal cells interactions, vasculature and the immune system – each of which contributes to tumor physiology. Given these considerations, *in vivo* animal models have provided the most clinically relevant experimental results to date (Figure 2C). Currently, mouse models of GBM are commonly used and include orthotopic xenografts of human, patient-derived GBM neurospheres, syngeneic transplants of mouse GBM cells and genetically engineered mouse (GEM) models. For a review, focused on *in vivo* models of GBM; please refer to [126].

In the last 10 years, orthotopic xenografts of patient-derived GBM neurospheres into immunodeficient mice have become the standard model for human GBM, in particular for evaluating efficacy of potential therapies [128,130]. Intracranial implantation of patient-derived neurospheres generates GBM tumors that recapitulate the invasive phenotype, histopathological features and genetic markers of the original patient [130,131]. Despite these advantages, the same caveats as described for neurosphere cultures – loss of tumor heterogeneity and possible phenotypic drift over long periods of culture – also apply to neurosphere-based xenografts [128]. While xenograft models enable the study of patient-specific tumors, they require that immunodeficient mice be used to prevent graft rejection. Typically used are nude or NOD-scid mice, which lack an adaptive immune response [129,131]. Because of this, important immunological events, such as interactions of GBM tumors with T cells, are not present [138,139].

Like others cancers, GBM tumors in different patients carry unique genomic aberrations that result in distinct therapeutic responses. Thus, development of strategies to identify effective, patient-specific therapies, otherwise known as personalized medicine, is a major goal of cancer researchers and clinicians. In the spirit of this goal, researchers have recently developed ‘AVATAR’ models of GBM patients, which involve direct orthotropic injection of fresh tumor cells from patients into NOD-scid mice within 12 h of tissue removal [140,141]. Unlike previous xenograft models, patient cells are never cultured *ex vivo*. AVATAR mice maintain genomic characteristics, subtype profile and histopathology of parental GBM better than patient-matched, neurosphere-based xenografts [140]. Furthermore, tumor formation and invasion in AVATAR mice directly correlate with patient outcomes [140]. In the future, AVATAR models may enable identification of patient-specific biomarkers that will more accurately predict clinical prognosis and treatment response. Despite improved fidelity of AVATAR models to clinical outcomes, there are still caveats. First, the molecular and functional differences between the murine and human brain microenvironments are significant. Second, the lack of a competent immune system in NOD-scid mice is a considerable source of variation from human patients, as described in the previous paragraph.

Immunocompetent models of GBM can be generated by syngeneic transplantation of mouse GBM cells into species-matched mice [129,139]. Models based on C57/Bl6-background mice have been widely used [139]. For example, a syngeneic model, where cells from the GL261 mouse GBM cell line are transplanted into C57/Bl6 mice, has become the ‘gold standard’ for studying immune cell–tumor interactions and therapeutic vaccines [139,142]. Despite their utility, syngeneic mouse models are not ideal, given the many differences in the physiology of mouse and human tumors [129]. Given the exciting success of immune-targeted therapies to treat cancers in recent years – in fact, multiple clinical trials for GBM immunotherapies are currently ongoing [143] – experimental models that better predict the human patient response to these therapies are needed.

A disadvantage of both xenograft and syngeneic models is that tumor initiation and development cannot be studied. To investigate these events, GEM models have been developed in which the role of specific gene mutations in tumor initiation and progression can be precisely studied [142,144]. Furthermore, targeted genetic manipulations – including mutations, silencing and overexpression – can be performed with temporal control [142,144–145]. As GEM models are created using immunocompetent mice, they can be used to study immune events that mediate tumor initiation. However, the GEM model that is faithful to human cancers and patient-specific features requires extensive understanding of roles of many cancer genes and do not account for differences between mouse and human immune systems [129]. In addition, a major drawback of GEM models is the inability to control tumor and the timing of tumor initiation, which hurts experimental reproducibility [139,144–145].

## Bioengineered, *ex vivo* models of GBM

While mouse models enable the study of GBM within the microenvironment of a living host, the cost, time, reproducibility and complexity of performing *in vivo* experiments present significant disadvantages. While *in vitro* culture systems can address these issues, experimental results often lack clinical relevance due to the absence of an appropriate microenvironment. Thus, researchers are actively working to develop advanced culture systems that accurately mimic the physical and chemical aspects of the native GBM microenvironment. The majority of *ex vivo*, biomimetic culture platforms developed to date involve hydrogel biomaterials – which exhibit tissue-like water content and mechanical properties, support 3D cell culture and can be fabricated from ECM-derived biomolecules. Although some progress has been made with controlled presentation of soluble factors to mimic *in vivo* microenvironments, at least at the time of this review, we have not found examples where these techniques have been applied to model GBM. Thus, this section emphasizes advancements achieving precisely and orthogonally altered ECM features within *ex vivo* culture platforms with the goal of creating a simplified context in which to make clinically relevant discoveries.

### 2D culture models

To add microenvironmental features to 2D cultures, researchers have cultured GBM cells on materials exhibiting stiffness closer to that of native brain and/or modified with ECM biomolecules using methods that provide greater use control and preservation of their native state than simple adsorption (Figure 2B). Many studies have employed polyacrylamide hydrogels [80,83–84,146–147], which can be readily modified to present varying mechanical properties [84,146], topographical structures [84] and bioactive molecules [80].

#### Mechanical influences in 2D culture

Whether caused by increases in ECM stiffness, interstitial pressure or both, it is generally thought that cells residing in or near GBM tumors experience stronger mechanical forces [77,80,87–88]. To explore the effects of these mechanical cues, researchers have cultured GBM cells on 2D substrates – fabricated from base materials including silicone rubber [148], polyacrylamide [80,83–84,146–147] and HA [28,85] – with varied mechanical properties. In the majority of these reports, more rigid substrates increased GBM cell migration, actin stress fiber formation and focal adhesion maturation. While the majority of these studies used glioma cell lines (e.g., U87MG and U373MG), O'Neill *et al*. [83] found that effects of stiffness on primary GBM cells were specific to the original patient – while some lines migrated faster on stiffer substrates, others were unaffected. Mechanical properties of the microenvironment likely also affect GBM cell proliferation. However, the few studies reporting these effects provide conflicting data [80,146,149]. These inconsistencies likely stem from a lack of independent controls for the effects of substrate stiffness and chemistry.

As discussed in the ‘Mechanical properties’ section, increased substrate stiffness correlates with increased activation of signaling pathways downstream of both integrins and EGFR [80]. Co-localization of focal adhesions with EGFR provided additional evidence of a coordinated mechanical response between EGFR and integrins. Mechanosensitivity of CD44 in GBM cells (U87MG and U373MG) has been demonstrated in 2D culture on HA-based biomaterials, where CD44 engagement mediated faster cell migration on substrates with increasing stiffness [28]. These types of culture systems provide the opportunity to study the mechanistic interactions among CD44, EGFR and integrins, which appear to drive GBM progression [18,28,44–45].

Physical topography and confinement of cells induce a response that is similar, although, perhaps, not identical to substrate mechanics [92]. Culture of transformed GBM cell lines in confined, micron-scale channels [84] or on substrates with aligned nanofibers [150,151] increased cell polarity and migration speed. Pathak and Kumar [84] used polyacrylamide to fabricate micron-sized channels with defined stiffness to decouple the effects of stiffness and confinement on migration of U373MG cells. Confined channels increased migration speed independently of stiffness, and a combined effect was observed, where cells in stiff, confined chambers migrated faster than those on unconfined substrates.

#### Effects of chemical composition of 2D culture

Nonbioactive 2D substrates, such as polyacrylamide or poly(ethylene glycol) (PEG), can be chemically modified to present bioactive molecules, including ECM-derived peptides and whole proteins, in a more controlled manner than simple adsorption [85,147,152]. Moreover, biomaterials presenting combinations of bioactive molecules can be used better to mimic the complex *in vivo* environment. HA-based hydrogels with covalently attached peptides or proteins have been used to investigate the interactive effects of CD44 and integrins on GBM cell behavior [28,80,85,153]. On 2D substrates, incorporation of HA directly increased migration speed of U87MG and U373MG cells [153]. 2D substrates fabricated from core–shell nanofibers, which provide control of mechanics and topography, can also be modified with ECM [150]. Core–shell nanofibers include a ‘core’, whose properties control substrate modulus, while the ‘shell’ can be independently modified to investigate specific effects of chemical coatings. For example, nanofibers with poly(ϵ-caprolactone) cores were modified with collagen, Matrigel or HA shells. In this system, motility of patient-derived GBM cells (OSU-2) on HA-shell nanofibers was slower than those with shells containing integrin-binding sites [150].

### 3D culture scaffolds

Although 2D cultures have provided valuable data, GBM cell behavior on these substrates does not necessarily reflect *in vivo* physiology. For example, the punctate focal adhesions that are often observed in 2D cultures [28,80,146] are not observed in 3D cultures [154] or whole tissues [155]. 3D cultures also better simulate diffusion of nutrients and oxygen through tissue and cell invasion through native ECM – both events dependent on scaffold pore size. In a 3D setting, the microenvironmental landscape directly affects diffusion of nutrients, metabolic waste and oxygen. In the GBM microenvironment, cells experience hypoxic conditions that further enhance malignant properties [156]. Thus, it is not surprising that 3D culture systems better preserve features of hypoxia-induced metabolism, treatment response and GSC phenotype (Figure 2C) [86,157–159]. Ability of cells to navigate through pores, degrade and remodel the scaffold also effects invasion [68–69,160–163]. Biomaterials designed to study GBM invasion are often prefabricated, cells seeded adjacent to or on a single side of the scaffold, and penetration of cells into and through the 3D scaffold observed [161,164–165]. Alternatively, cells are first cultured in ‘hanging droplets’ then surrounded by a 3D scaffold into which they can invade [86,149,153]. Encapsulation of GBM cells within a 3D hydrogel microenvironment – where highly biocompatible crosslinking chemistries are used to form the scaffold around live cells – is also common (Table 1) [44–45,158–160,162,166–167].

**Table 1.** **Hydrogels used as 3D cell culture scaffolds to mimic the glioblastoma microenvironment.**

**Hydrogel base material**	**Crosslink method and functional groups**	**Factors included**	**Dimensionality**	**Notable findings**	**Human GBM cell lines**	**Ref.**
***Covalent crosslinks***						
Polyacrylamide	Acrylamide, bisacrylamide	None	2D	Increasing substrate stiffness and confinement increased cell migration	U373MG	[84]
HA	Condensation/adipic dihydrazide, COOH	κE	3D	κE increased migration and secretion of MMP-2 and MMP-12	CB74, CB109, CB191	[164]
HA	Michael addition/thiol, methacrylate	Fibronectin	2D	Increasing stiffness and fibronectin content increased migration	U373MG	[85]
HA	Michael addition/thiol, diacrylate, and thiol, divinyl sulfone	Gelatin, HGF	3D	Increasing stiffness reduced migration distance. HGF increased migration	U118, U87R	[149]
HA	Michael addition/SH-acrylate	None	2D, 3D	3D encapsulation of cells in increased their radio- and chemoresistance	U87MG, primary cells isolated from seven different patient tumors	[159]
***Noncovalent crosslinks***						
Alginate	Ca^2+^ mediated	RGD peptides	3D	Cells were more susceptible to toxins in softer hydrogels	U87, U51	[82]
Collagen I	Phase transition	EGF	2D, 3D	In 3D, EGF increased directional persistence of migrating cells	U87-MG	[168]
Collagens I, III and IV	Phase transition	HA	3D	Cell morphology depended on collagen type. Higher HA concentration limited migration	OSU-2 (patient-derived)	[167]
***Photo-crosslinks***						
Polyacrylamide	Chain growth/acrylamide, bisacrylamide	None	2D	Increasing substrate stiffness and confinement increased cell migration	U373MG	[84]
HA	Chain growth/methacrylate	None	3D	Increasing HA concentration reduced proliferation	U87MG	[45]
PEG	Chain growth/thiol, acrylate	HA	2D, 3D	Inclusion of HA increased oncogenic markers	U87MG	[44]
PEG	Step growth/thiol, norbornene	HA, MMP-degradable sites	3D	Increasing concentration of MMP-degradable sites promoted cell migration	U87	[163]

GBM: Glioblastoma; HA: Hyaluronic acid; MMP: Matrix metalloprotease; PEG: Poly(ethylene glycol).

#### Fabrication of scaffolds for 3D culture

Given that cell–cell and cell–ECM interactions in GBM are highly complex and heterogeneous, *ex vivo* culture platforms in which individual aspects of the microenvironment are isolated experimentally will enable researchers to ‘tease apart’ these entangled effects in a simplified context. This section provides a basic overview of the tools available to create 3D, bioengineered scaffolds, while the following sections discuss how researchers have applied these tools to study GBM. Although 3D scaffolds can be fabricated from macroporous, solid plastics, such as poly(lactide-co-glycolide), these materials require that cells be seeded on top and enter scaffolds either passively by gravity or actively by migration [114,158]. In contrast, hydrogels are more similar to the native brain microenvironment in water content and mechanical properties. Hydrogels are ideal for studying GBM physiology because they can be formed using gentle, aqueous chemistries to encapsulate live cells; provide constant, insoluble cues from native ECM components (as opposed to denatured, adsorbed proteins); and permit observations of 3D cell migration and matrix remodeling. Hydrogels are typically transparent, enabling facile imagine of live cells in 3D culture using standard optical techniques and better preserve *in vivo* physiology of encapsulated GBM cells than traditional culture methods [159]. Although many of the methods for hydrogel fabrication discussed in the following paragraphs have not yet been applied to study GBM, they provide invaluable tools for creating biomimetic, *ex vivo* models in the future

Hydrogels are formed by covalent or physical (noncovalent) crosslinking of hydrophilic polymer chains into insoluble networks using a variety of chemical methods. Covalent crosslinking requires functional groups that react either spontaneously when in proximity with a complementary moiety or when activated by an initiator. Condensation, Michael-type addition and Diels–Alder reactions are widely used to fabricate hydrogels for 3D cell culture, because they proceed readily under physiological conditions. Condensation reactions are defined by the formation of a small molecular by-product, typically H_2_O. Biocompatible hydrogels are often formed via condensation reactions between amines (NH_2_) and carboxylic acids (COOH) [164,169]. Many biocompatible hydrogels are crosslinked via Michael-type addition between a thiol (SH) and an acrylate or vinyl sulfone – forming thioester or thioester bonds, respectively [28,85,147,149,153,159,167].

Since crosslinking does not occur until exposure to light, photochemistries can be used to generate hydrogels with precise spatial and temporal controls. When modeling GBM, photochemical patterning is an attractive strategy for creating gradients of microenvironmental features. Hydrogels are often photo-crosslinked through chain-growth polymerization of acrylates [44,45]. More recently, thiol–ene photoreactions (e.g., between thiol and norbonene groups) have gained popularity [163,166]. Thiol–ene reactions proceed by step-growth polymerization, which yield hydrogels with more defined networks and fewer defects than those produced by chain-growth polymerization [170]. Because of its relatively high water solubility and biocompatibility, the UV-activated radical initiator, Irgacure^®^ 2959 (also known as, 1-[4-(2-hydroxyethoxy)-phenyl]-2-hydroxy-2-methyl-1-propane-1-one) has been commonly used to fabricate biocompatible hydrogels. However, a few recent studies have used phenyl-2,4,6-trimethylbenzoylphosphinate as a more biocompatible alternative, with higher water solubility and more efficient photoactivation than Irgacure 2959 [171]. Use of phenyl-2,4,6-trimethylbenzoylphosphinate will likely enable 3D cultures of sensitize, primary GBM cells within photo-crosslinked hydrogels.

Noncovalent crosslinks are typically formed via temperature or pH-induced segregation of polymer regions based on hydrophobicity or ionic interactions. Although resultant hydrogels are often weaker than those formed by covalent crosslinks, noncovalent gelation methods are usually more biocompatible. Noncovalently crosslinked hydrogels commonly used for 3D culture include collagen I [86,162,167–168,172] and laminin I-based Matrigel, which both undergo gelation at physiological temperature. Alginate hydrogels, which are crosslinked via divalent cations such as Ca^2+^, are also popular [82,165].

#### Effects of stiffness in 3D culture

**Figure 3.**
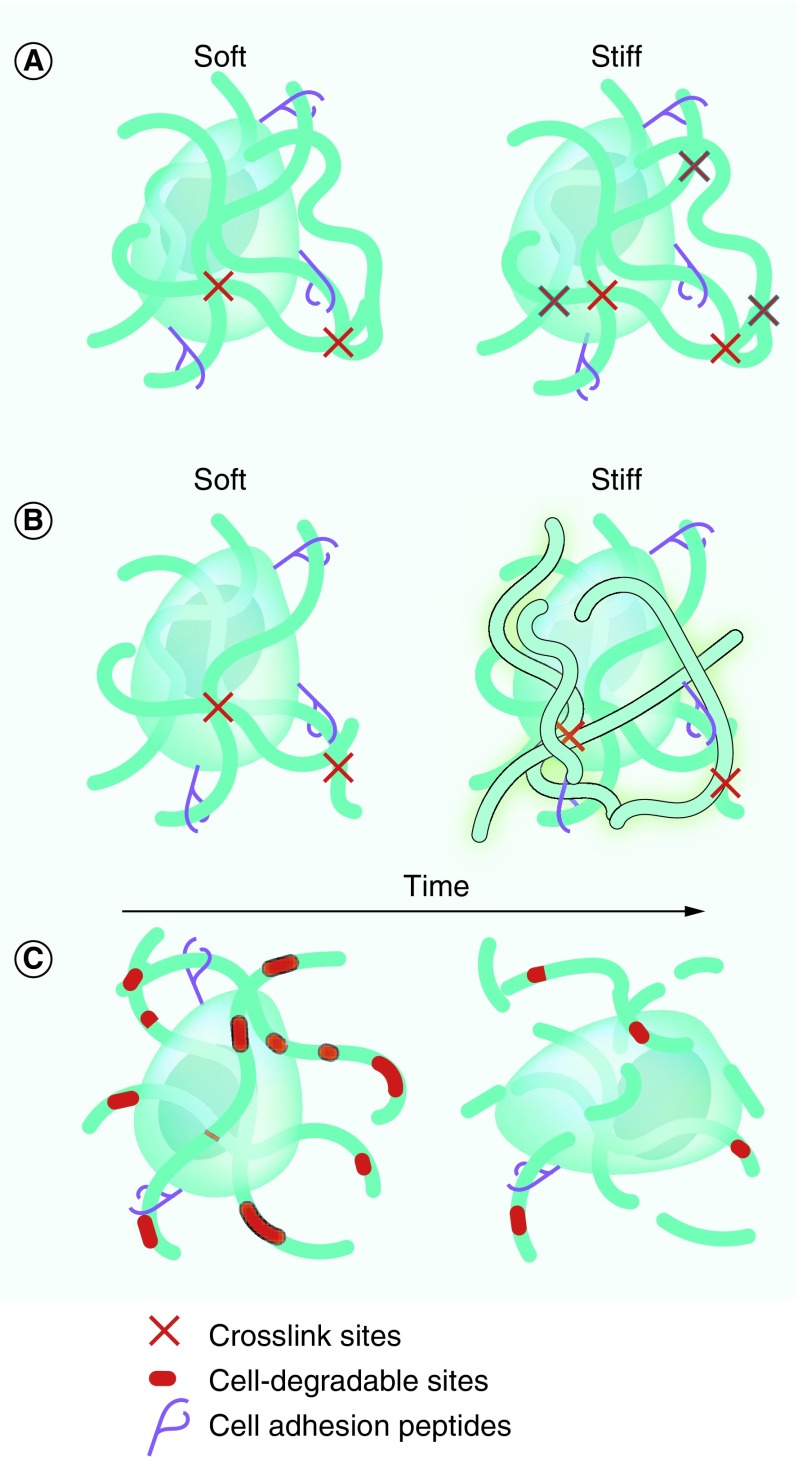
**Controlling biochemical and physical properties in 3D hydrogel biomaterials.** Mechanical properties can be tuned by **(A)** altering crosslink density or **(B)** base polymer concentration, both of which affect hydrogel pore size and diffusion of soluble factors through scaffolds. **(C)** Incorporation of degradable polymers, such as matrix metalloprotease- or hyaluronidase-susceptible sites, facilitates cell migration and degrades scaffolds over time.

As described in the ‘Mechanical properties’ section, GBM behavior is regulated by the mechanical properties of the surrounding microenvironment. To study this effect *ex vivo*, it is important to control the mechanical properties of 3D scaffolds. In general, mechanical strength of hydrogel scaffolds increases with the crosslinking density (Figure 3A) or the concentration of the backbone polymer (Figure 3B). In Michael-type addition hydrogels, changing the molar ratio of the donor (e.g., thiol) to acceptor (e.g., vinyl sulfone) groups can be used to alter the density of crosslinks and thus the mechanical properties. In photo-crosslinked hydrogels, by increasing the number of reactive groups (e.g., acrylates), initiator concentration or light exposure will yield higher mechanical moduli. Similarly, in noncovalently crosslinked alginate hydrogels, increasing the Ca^2+^ concentration results in more crosslinks and stiffer hydrogels.

In contrast to 2D cultures, GBM cells (U87MG, U87R, U118, U373MG and U251MG) encapsulated in 3D matrices increase migration speed with decreasing scaffold stiffness [86,149,153]. However, conflicting results have been reported as to how mechanics affect MMP secretion. For example, culturing U87MG GBM cells in HA-based hydrogels with increasing stiffness is reported to both increase [44,45] and decrease the [166] MMP-9 production. As with 2D cultures, there are discrepancies as to how scaffold stiffness affects GBM cell (U87MG and U118) proliferation in 3D culture [45,149,166]. Many of these discrepancies may be caused by an inability to decouple effects of mechanical features from other extracellular cues.

When using bioactive molecules, such as HA or collagen, as the hydrogel base, controlling scaffold mechanics via polymer concentration means that the biochemical properties are also affected (Figure 3B). As each of these properties has independent effects on GBM cell behavior, it is important that they can be experimentally decoupled. Alternatively, the crosslinking density can be used to alter mechanical properties – for example, by increasing the initiator concentration of when photo-crosslinking – without changing the base polymer concentration (Figure 3A). However, changes to either crosslinking density or polymer concentration can significantly affect pore size, and thus diffusion through hydrogel scaffolds. It is likely that many discrepancies concerning effects of scaffold stiffness in 3D cultures may stem from an inability to decouple stiffness from other microenvironmental cues, such as biochemical composition.

#### Effects of biochemical interactions in 3D culture

To mimic the biochemical composition of the native brain and GBM tumor microenvironment, 3D hydrogel scaffolds have been fabricated from a variety of ECM-derived biopolymers, including HA [44–45,147,163–164,172], chitosan [158,165] and chondroitin sulfate [167] polysaccharides, and collagen/gelatin proteins [44–45,157,162,167,172]. As the ECM in the CNS contains high amounts of GAGs and few fibrous proteins like collagen I, many researchers have used HA-based hydrogels to mimic native brain. To enable crosslinking, HA and other GAGs require chemical functionalization with crosslinking moieties such as thiols or acrylates. In these hydrogels, increasing amounts of both HA and/or chondroitin sulfate GAGs enhanced GBM cell (U87MG, U251MG and U373MG) migration [153,161,169]. In addition, higher HA content is reported to decrease proliferation (U87MG) [45] and increase the expression of genes associated with GBM progression, including HA synthases (patient-derived, GSC11) [172], hyaluronidases (patient-derived, GSC11) [172], MMP-9 (U87MG) [44,45], MMP-2 (U87MG) [45], VEGF (U87MG) [44,45] and HIF-1 (U87MG) [44,45]. In addition, cultures of multiple patient-derived GBM lines in 3D HA hydrogels better approximate resistance to radiation and chemotherapy observed in clinical tumors [159].

As HA is not generally cell adhesive, gelatin (modified to permit crosslinking with groups such as acrylates) is sometimes added to provide sites for integrin attachment [44,45]. Increasing gelatin concentration in HA-based hydrogels upregulated MMP-9, VEGF, HIF1 and fibronectin expression, while downregulating MMP-2 in U87MG cells [44,45]. While gelation (denatured collagen) typically requires chemical modification for crosslinking, noncovalently crosslinked hydrogels of collagen I form spontaneously under physiological conditions through fibrillogenesis.

Hybrid scaffolds of HA and collagen I have also been used to investigate the effects of HA on migration of a few patient-derived GBM neurosphere lines [167,172]. In one study, interpenetrating networks – where hydrogels of collagen I hydrogels were infused with thiol-modified HA crosslinked through disulfide bonds – were used to evaluate effects of HA content and found that adhesion and migration speed of GBM cells (OSU-2) decreased with increasing HA concentration [167]. In contrast, a more recent study found that addition of HA to collagen I hydrogels facilitates migration (GSC11 cells) [172]. Although high molecular weight HA (>250 kDa) was used in both studies, in the latter HA remained unmodified and uncrosslinked, and, instead, was used to simply coat colIagen I fibers [172]. As collagen I is not typically present in the brain [3,173], hydrogels created using this method likely better recapitulate the ECM of peripheral tissues. Furthermore, modification of the HA backbone – especially the carboxylic acid site on glucuronic acid – can disrupt the hydrogen bond-dependent 3D structure of HA and interfere with cell receptor interactions [174]. As the 3D structure of high molecular weight HA is at least partially responsible for its ability to induce different cell responses than its low molecular weight counterparts, it is possible that the high degree of thiolation used in the former study altered HA bioactivity [167,175]. The latter study also reported that expression of CD44 and CD168 receptors and production of new HA in GBM cells increased with HA content, implying that HA–receptor interactions were also enhanced [172]. Finally, the latter study cultured GBM cells as spherical aggregates [174], rather than dissociated single cells [167]. Culture in aggregated ‘microtissues’ is more reflective of the native GBM microenvironment because it permits direct cell–cell contacts and collective migration of cells from a central source.

Nonadhesive PEG [163,166] and alginate [82,165] hydrogels have been used as ‘blank slates’ to which bioactive molecules are added. As proteins and peptides naturally contain cysteines, they are often tethered to hydrogel backbones through thiol-based chemistries, such as Michael-type addition [158,163,176]. Using this method, a specified number of arms of branched PEG macromers can be modified with bioactive molecules, so that a defined number of arms are left to participate in hydrogel crosslinking [176]. Multiarm, branched PEG macromers yield more defined networks than linear PEGs, as network formation does not rely on chain entanglement [176]. Alternatively, photochemical means are used to conjugate bioactive molecules into crosslinked networks. For example, acrylate-modified biomolecules can be mixed into PEG-acrylate solutions prior to activation of a photoinitiator to induce hydrogel formation [44–45,171].

In addition to whole proteins, such as fibronectin [85], ECM-derived adhesive peptides, such as those containing the ubiquitous integrin-binding RGD sequence [28,82,153,163,166], are commonly used. Although integrin-binding peptides may not have the comprehensive effects of their full-length counterparts, they can isolate effects of matrix interactions with specific types of integrins. Furthermore, it is generally easier to control their functional presentation within hydrogel matrices. When incorporated into HA-based hydrogels, RGD peptides increase GBM cell (U87MG and U373MG) adhesion [153]. Conjugation of RGD peptides to nonadhesive alginate hydrogels has been shown to provide protection against toxin-induced apoptosis of GBM cells (U87 and U51) [82]. Other ECM-derived adhesive peptides have also been explored. For example, functionalization of HA hydrogels with a κ-elastin-derived peptide increased invasion and production of MMP-2 and MMP-12 in multiple patient-derived GBM cell lines [164].

Growth factors and cytokines can be incorporated into scaffold microenvironments through simple diffusion of solubilized factors, direct chemical conjugation or noncovalent tethering to the hydrogel matrix. Overexpression of EGFR in GBM cell lines, in combination with 3D culture in HA–gelatin hydrogels, upregulated GBM cell (U87MG) expression of MMP-2 and MMP-9 [44,45]. Furthermore, while addition of soluble EGF to 3D collagen hydrogel cultures increased directional persistence of migrating U87MG cells, it had the opposite effect in 2D cultures on collagen substrates [160]. In the native ECM, noncovalent interactions of diffusible growth factors with heparins both increase potency and create bioactive concentration gradients of cytokines [36]. Hydrogel scaffolds have been modified with heparin to mimic this phenomenon in *ex vivo* microenvironments [177].

#### Biomaterial microenvironments to model 3D GBM invasion

In tissues, migration requires that cells either squeeze through open pores or degrade surrounding ECM to create a path. Unlike 2D substrates, 3D matrices can model these crucial aspects of GBM invasion *ex vivo*. However, in the majority of 3D experimental models of GBM, the effects of polymer stiffness, ECM concentration and porosity cannot be decoupled. For example, stiffer scaffolds are often fabricated by increasing the concentration of the polymer backbone and/or the density of crosslinks – both of which decrease pore size (Figure 3A & B). Hydrogels fabricated from ECM components yield bioactive scaffolds that mediate cell migration not only through adhesion, but also through their capacity to be degraded and remodeled by migrating cells (Figure 3C) [161,162]. In this case, increasing base biopolymer concentration also affects the number of cell adhesive sites and density of degradable ECM. The inability to decouple scaffold mechanics, porosity, adhesivity and degradability likely contributes to many of the discrepancies in reports of their individual effects on GBM invasion.

In 3D collagen hydrogels, nanofibers can provide a physical structure to guide migrating cells. Migrating cells also produce MMPs to degrade collagen-I hydrogels. Notably, one report found that U87MG cell migration in 3D collagen scaffolds was dependent on the expression of MMP-1, while migration on 2D collagen fiber mats was independent of MMP production [159]. Faster migration of patient-derived GSCs was induced by doping collagen scaffolds with tenascin-C through its degradation by MMP-12 [162]. Despite the widespread use of collagen scaffolds to study cell migration [160–161,172], it is not clear how relevant these results are to GBM physiology, as the brain ECM has negligible amounts of fibrous collagen [3,173].

As detailed in the ‘Extracellular matrix degradation’ section, a major pathological feature of invasive GBM is MMP overexpression. To create controlled models of GBM cell migration in 3D scaffolds, researchers have incorporated MMP-degradable sites into hydrogel crosslinks (Figure 3C). Often, this is accomplished by reacting MMP-degradable peptides functionalized with cysteine-bearing thiols at either end with a backbone polymer bearing thiol-reactive moieties, like acrylates or vinyl sulfones. Incorporation of MMP-cleavable sites into nondegradable PEG hydrogels greatly improves the ability of U87 cells to infiltrate 3D scaffolds [162,165]. ECM polysaccharides, such as HA, are also degraded by cell-produced hyaluronidase, which is overexpressed in clinical GBM tumors [13], and facilitate migration of GBM cells (U87MG, U373MG, U118, CB74, CB190 and CB191) through HA-containing hydrogels [149,153,161,164,172]. HA can also indirectly facilitate U87 cell invasion by upregulating production of various protein-targeting MMPs [44–45,172].

Only a handful of biomaterial platforms that allow for some degree of decoupling of mechanical properties, porosity and/or biochemical cues have been used for 3D GBM cell culture [45,86]. Kumar and the co-workers [86] fabricated interpenetrating hydrogel networks of collagen I and agarose – where increasing the concentration of nonadhesive agarose was used to create stiffer scaffolds while keeping collagen I levels constant – and found that softer hydrogels promoted GBM cell (U87MG, U373MG and U251MG) migration. Although this system effectively decoupled stiffness from matrix adhesion and degradability, increased agarose density may have result in smaller pores that hinder migration in stiffer hydrogels. Although not yet used to study GBM migration, 3D scaffolds created from PEG-based ‘microribbons’ have been developed in which stiffness and macroporosity are decoupled [178]. The use of covalently adaptable networks could potentially circumvent the need for orthogonal control of substrate stiffness in experimental models of cell migration [179]. In these networks, crosslinks can be broken and re-formed dynamically, permitting relatively unhindered cell migration through even nanoscale-sized pores without active matrix degradation.

Gradients of microenvironment properties, including stiffness, ECM content and soluble factors, can all drive cell migration. Pedron *et al*. [45] developed hydrogel platforms with gradients of stiffness/crosslinking density, HA content and cell concentration using a microfluidic platform demonstrated its utility to study the effects of these gradients on U87MG cell migration. Kumar and the co-workers [85] developed an innovative platform in which gradients of stiffness were created independently of gradients of ECM proteins in a single hydrogel. This was accomplished by using orthogonal photochemistries – where crosslinks were added to hydrogel networks in response to visible light exposure to control stiffness while fibronectin immobilization was triggered by UV light. Although these platforms represent an important technological advance, hydrogel stiffness is still coupled to crosslinking density and pore size. Park and Gerecht [180] recently developed a novel method by which to create hydrogel cultures with defined hypoxic gradients. Although not yet used to study GBM, these hydrogels were used to demonstrate how controlled, quantitative variation of hypoxic gradients influences sarcoma cell invasion [181]. In future studies, such strategies could be combined with more established methods to control ECM and mechanical aspects of the GBM microenvironment.

#### Modeling cell–cell interactions in 3D culure

To investigate reciprocal interactions of GBM cells with other cell types that are present in the *in vivo* microenvironment – including astrocytes, microglia/macrophages and endothelial cells – researchers have worked to develop coculture models to study the effects of both paracrine signaling and direct cell–cell contacts Figure 1B). While several previous studies have investigated the effects of cell-produced factors of nontumor cells on GBM cells cultured on 2D substrates using conditioned media, these experiments cannot provide information about crosstalk between cell types. Transwell [182,183] and Boyden chamber [47] assays have been used to investigate crosstalk through paracrine signaling, but cannot easily determine effects of direct cell–cell (juxtracrine) interactions or those with secondary structures, such as blood vessels. In the native GBM microenvironment, cells interact with each other in 3D through direct contacts and diffusing paracrine factors, and thus 3D culture models are necessary to recapitulate these interactions.

To study the angiogenic effects of GBM cells, a transformed endothelial cell line (HUVEC) was cultured on dextran beads and then embedded into a 3D fibrin hydrogel scaffold [184]. Culture of transformed GBM lines (U87 and LN18) on the top surface of HUVEC-embedded, fibrin hydrogels increased angiogenesis [184]. However, this method did not allow for direct contact of GBM and endothelial cells in three dimensions. A more recent study overcame this limitation through coculture of transformed GBM lines (U251 and LN18) with an immortalized astrocyte line (TNC-1) within 3D spheroids to demonstrate how the presence of astrocyte protected GBM cells from temozolomide-mediated apoptosis [185]. Segall and the co-workers [186] have developed a method for 3D coculture of GBM (mouse GL251, human U87) and microglia/macrophage (THP-1) immortalized lines, where cell mixtures are embedded into a Matrigel scaffold that can be used to investigate effects of both paracrine and juxtracrine interactions on GBM cell invasion.

Despite these improvements to 3D cocultures methods, we have found no examples of cocultures with patient-derived GBM cells at the time of this review. One challenge to developing cocultures with patient-derived lines is that serum cannot be used without altering GBM cell phenotype [187]. However, serum is often required to maintain cultures of astrocyte and endothelial cells. cocultures with immune cells and astrocytes can also be difficult to interpret, as culture conditions may promote inflammatory phenotypes (e.g., GFAP expression in astrocytes on hard, 2D substrates [188]) that are not reflective of their GBM-associated counterparts *in vivo*. In the future, methods for defined, mixed cultures of different GBM cell subtypes (e.g., GSCs) found in heterogeneous tumors would also be valuable – however, identifying culture conditions (in particular, media formulations) that maintain these separate phenotypes and can isolate their individual effects remains a formidable challenge. As an alternative to true cocultures, synthetic peptides that mimic juxtracrine receptors, such as N-cadherin, can be incorporated into biomaterials [189]. Although this method cannot be used to characterize dynamic crosstalk between live cells, it provides a simpler method to investigate the isolated effects of cell–cell contacts.

To enable *ex vivo* investigations of interactions of GBM tumors with bloods vessels – for example, to study effects of vessel guidance on migration – cocultures of actual vessels formed from endothelial cells (and ideally including pericytes) will be required. Recently developed ‘organ-on-chip’ devices, such as the AngioChip^®^ may provide the technology to perform these types of study [190]. ‘Organ-on-chip’ astrocyte–endothelial cell cocultures that model the *in vivo* BBB may also be used in the near future to study the interactions of GBM tumors with vasculature structures [191,192].

## Conclusion & future perspective

The most important metric of the value of experimental GBM models is their ability to accurately reflect and predict patient outcomes. Although many improvements have been made over the past 10–15 years (Figure 2), many models still do not adequately accomplish this primary objective. The combination of improved methods for maintaining tumor-specific heterogeneity and physiology in cultured cells, and engineering biomaterials as defined, 3D culture environments provide an exciting path forward to achieve efficient, clinically accurate models of GBM that lead to more effective therapies.

The cells used for any model are crucial to its clinical relevance. Use of patient-derived cells cultured as neurospheres represents a significant improvement over transformed glioma cell lines. However, during neurosphere culture – prior to orthotopic transplantation into a mouse or 3D culture in a biomaterial – these cells lose heterogeneity and, as such, do not accurately reflect the original tumor composition. Recently developed methods for generating GBM organoids [137] and AVATAR mouse models [140,141] each appear to significantly improve preservation of tumor heterogeneity and clinically relevant physiology. One plausible explanation for GSC enrichment – whose survival tends to be adhesion independent – within patient-derived neurospheres is the lack of an adhesive matrix. In contrast, AVATAR models – created by orthotopic transplantation of freshly dissociated patient tissue – bypass neurosphere culture and, instead, immediately provide crucial microenvironmental support. This likely promotes survival of more adhesion-dependent cells present in patient tumors to better preserve heterogeneity. As opposed to animal-based models, biomaterial systems avoid introduction of nonhuman components and are more reproducible. Direct transplantation of biopsied cells into biomaterial microenvironments engineered to preserve heterogeneity and physiology of patient tumors presents an attractive alternative to animal models.

Furthermore, biomaterial models provide exciting opportunities for personalized medicine. While organoid, xenograft and AVATAR models require weeks to months to establish, biomaterial cultures can be established within days to weeks. Despite the impressive ability of AVATARs to predict patient-specific outcomes [140,141], the extended period of time required to make these predictions prevents any results from being used to treat a particular patient. While AVATARs appear excellent models for subtype-specific GBM tumors, treatment of patients based on these models does not necessarily improve patient outcomes [141]. Given the fast time to establish patient-specific cultures, uniform and controlled fabrication and affordability of biomaterial microenvironments compared with animal models, it is feasible to perform high-throughput, parallel screens of various treatments to potentially identify and implement patient-specific treatment strategies within a clinically relevant time frame.

Engineered, biomaterial-based models provide an *ex vivo* experimental platform in which the direct effects of individual microenvironmental parameters on GBM physiology can be studied in a controlled context. Given the complexity of the brain microenvironment, it is often challenging to isolate the contribution of any individual feature to a particular aspect of GBM physiology. Through controlled variation of microenvironmental components, biomaterial platforms can potentially isolate these effects and identify specific therapeutic targets. Robust methods to independently control the presentation of individual features of the GBM microenvironment in a single biomaterial platform would greatly improve the ability of such models to accurately predict clinical outcomes. Researchers are also working to add complexity to biomaterial microenvironments to better mimic the GBM microenvironment, for example, by developing coculture systems with nontumor cells [114,122] and methods to generate orthogonal gradients of bioactive features [45,85].

As artificial GBM microenvironments have not yet achieved the ability to replace the need for *in vivo* models, it is important to ask how much complexity and which specific features are minimally required to achieve a physiologically relevant model *ex vivo*? Answering this question will require rigorous evaluation of model fidelity to clinical cases. To achieve this, biomaterial studies will need to include cells from clinically relevant sources, such as patient-derived neurospheres or even freshly biopsied cells. Given the variability across patient samples and GBM subtypes, inclusion of parallel experiments using multiple patient-derived lines would further improve the physiological relevance of results. Although the majority of biomaterial-based studies have focused on invasion, future studies should include equally important GBM tumor features, such as treatment resistance. Finally, model validation will likely require parallel comparisons 3D cultures with patient outcomes (through histopathological analyses of biopsied tumors and resources such as The Cancer Genome Atlas) and mouse xenografts. Ideally, cells from the same patient would be studied *ex vivo* in biomaterials and *in vivo* as orthotopic transplants and results compared with patient-matched clinical data.

**Executive summary**

- Glioblastoma (GBM) is an extremely lethal brain cancer, with rapid progression and high rates of recurrence.
- The microenvironment of GBM tumors drives invasion and resistance to treatment.
- Current experimental models of GBM do not adequately reflect patient outcomes, presenting a challenge to the development of improved treatments.
- Biomaterial microenvironments can be engineered to mimic multiple aspects of the GBM microenvironment in a controlled manner.
- Biomaterials can be used as clinically relevant models of GBM to better understand the specific effects of individual features of the microenvironment on tumor physiology and develop new, more effective treatments.
